# Multifunctional Thermoplastic Paper Enabled by Plant‐Cell‐Derived Additives: A Paradigm of Paper‐Based “Modern Alchemy”

**DOI:** 10.1002/advs.202506157

**Published:** 2025-11-05

**Authors:** Xiaoyan Yu, Jie Zhou, Jianqiang Li, Hongyang Yuan, Xueren Qian, Yonghao Ni, Zhibin He, Chaoji Chen, Jing Shen

**Affiliations:** ^1^ Research Division for Sustainable Papermaking & Advanced Materials, Key Laboratory of Biobased Materials Science and Technology (Ministry of Education) Northeast Forestry University 26 Hexing Road Harbin 150040 China; ^2^ School of Resource and Environmental Sciences, Hubei Biomass‐Resource Chemistry and Environmental Biotechnology Key Laboratory Wuhan University Wuhan 430079 China; ^3^ Engineering Research Center for Hemp and Product in Cold Region of Ministry of Education, School of Light Industry and Textile Qiqihar University Qiqihar 161006 China; ^4^ Shandong Huatai Paper Co., Ltd. Dongying 257335 China; ^5^ Limerick Pulp and Paper Centre, Department of Chemical Engineering University of New Brunswick Fredericton NB E3B 6C2 Canada; ^6^ Department of Chemical and Biomedical Engineering University of Maine Orono ME 04469 USA

**Keywords:** biomass, bioplastics, cellulosic materials, paper‐based advanced materials, pulp and paper industry, papermaking wet‐end chemistry and chemical additives

## Abstract

Papermaking, an ancient yet remarkable invention, hinges on the formation of a network of plant cells. With the growing demand for bio‐derived alternatives to non‐renewable resources and difficult‐to‐degrade plastics, enhancing the functional attributes of cellulosic paper is essential to broaden its applications. Here, a facile approach is introduced to upgrade conventional cellulosic paper into an advanced thermoplastic biomaterial, endowed with ductility, wet‐strength, gas and liquid barrier functionalities, and antistatic properties. The concept is grounded in the specialized area of papermaking wet‐end chemistry and chemical additives and employs plant‐cell‐derived cellulosic additives, prepared via ring‐opening‐based heterogenous chemical engineering of paper‐grade pulp with microstructurally porous cell walls comprising fibrils, which are then formed upon dissolution in an aqueous “non‐derivatizing” solvent, for engineering the paper through a process that somehow mimics the industrial surface sizing. The utilization of additives initiates a form of paper‐based “modern alchemy”, which involves the encapsulation of fibers with ring‐opening‐engineered cellulosic structures, solvent‐induced fiber annealing, bridging of interfiber gaps, film‐forming, porosity reduction, structural densification, enhanced internal bonding, paper surface smoothening, etc. The engineered paper can be facilely reshaped through hot‐pressing for 3D forming and recyclable applications. Additionally, their dissolution in a cellulosic solution yields functional additives for diverse applications, offering another avenue for recycling. This work offers insights into designing paper‐based thermoplastic materials using sustainable additives.

## Graphical abstract

Building upon the concepts of papermaking wet‐end chemistry and chemical additives, this work upgrades conventional cellulosic paper into thermoplastic material using plant‐cell‐derived additives, synthesized via ring‐opening modification of pulp fibers and further disassembly into aqueous solutions as additives. Applied through surface engineering, the additives induce fiber encapsulation, annealing, densification, and enable reshaping, recyclability, and sustainable reuse.

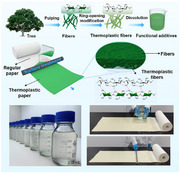

## 1 Introduction

Sustainable materials derived from renewable resources are essential for shaping a greener future.^[^ ^1^ ^]^ Paper is one of the oldest bio‐derived sustainable materials with attractive features such as flexibility, low cost, and light weight. The processes of pulping and papermaking involve the processing of naturally‐bonded plant cells (predominately with high aspect ratios, also known as fibers) into versatile materials for writing, printing, and packaging, among other uses. Structurally, paper is a hydrogen‐bonded network of plant cells, and this network is a reshaped structure of natural cellulosic materials such as wood, bamboo, and rice straw.^[^ ^2^ ^]^ Notably, paper possesses intrinsically sustainable features, including the use of renewable feedstocks, easy recyclability, utilization of water as a nontoxic and recyclable medium, and facile biodegradability, setting it apart as a distinctive ecofriendly material.

However, a need remains for a more sustainable pulp and paper industry. The current energy‐consuming nature of unit operations is one of the significant challenges.^[^ ^3^ ^]^ Technologies involving reduced consumption of energy and water, integrated lignocellulosic biorefinery, waste and contaminant treatment, capture of gaseous emissions, sustainable forest management, alternative feedstocks, waste paper recovering and recycling, sustainable energy, and green chemical additives are essential for enhancing the sustainability of the industry. Encouragingly, bio‐derived additives such as those derived from starch have been widely used to tailor paper properties since ancient times. The wet‐end and surface applications of water‐soluble biopolymers are common industrial practices. Fossil‐derived additives, however, are still required in certain cases, covering a wide spectrum of applications such as wet‐strength development, binding pigment particles to paper, and delivering liquid‐barrier properties. An example of such fossil‐derived additives are synthetic polymers such as polyamidoamine epichlorohydrin and cationic polyacrylamide. In the specialized area of papermaking wet‐end chemistry and chemical additives,^[^ ^4^, ^5^ ^]^ the flexible and tailored use of chemical additives for wet‐end and non‐wet‐end applications holds great promise, and indeed, there remains a need to examine the possibilities of designing bio‐derived additives for greener processes and more sustainable products.

Designing sustainable materials like cellulosic paper for the ever‐broadening scope of uses and applications holds great potential, particularly in the context of ever‐growing awareness of problematic, difficult‐to‐degrade plastics.^[^ ^6^, ^7^, ^8^ ^]^ Despite this potential, the specialized or unconventional applications of conventional paper products are challenged by the limitations in thermoplasticity, barrier properties, ductility, wet strength, etc.^[^ ^9^, ^10^ ^]^ Combining functional materials with cellulosic paper to form composites enables various applications. Paper‐based functional composites are promising in advanced applications: energy storage, health diagnostics, environmental monitoring, ink‐free printing, and food quality testing, among many others.^[^ ^11^, ^12^, ^13^, ^14^, ^15^, ^16^, ^17^, ^18^, ^19^ ^]^ It is also worth noting that, besides plant cells that are essentially known as fibers, paper is also producible from animal fibers, mineral fibers, synthetic polymeric fibers, and other materials (e.g., graphene and graphene oxide).^[^ ^20^, ^21^ ^]^ Furthermore, the use of chemical additives to transform cellulosic paper into a plastic‐like material^[^ ^22^ ^]^ are promising as it aligns with paper production processes as post‐treatments. Nevertheless, there is an ongoing need to design paper‐based advanced materials for a wide range of applications, particularly by incorporating green and sustainable additives.^[^ ^23^ ^]^

**Figure 1:**
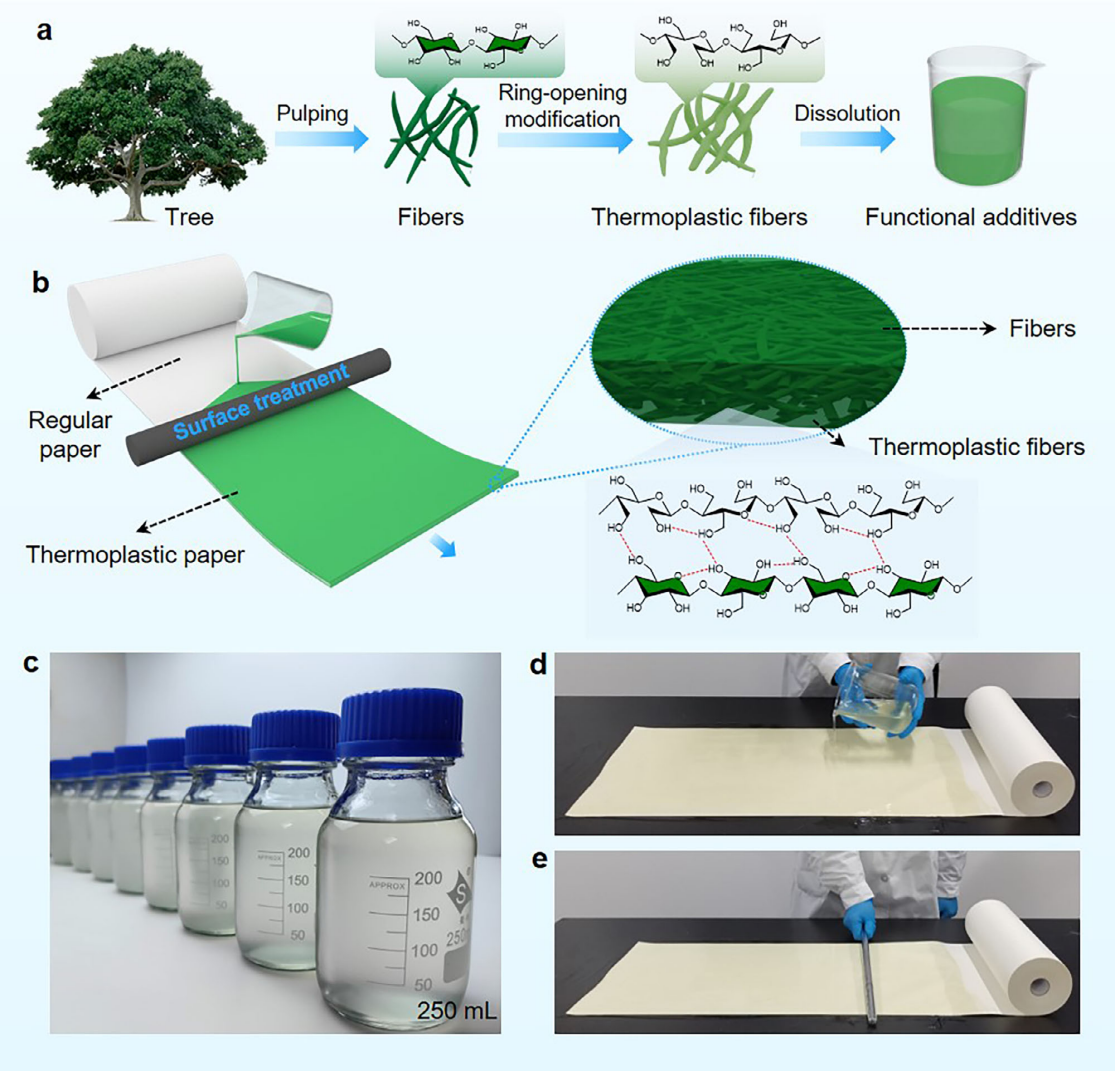
Conceptual illustration demonstrating the utilization of liberated cellulosic plant cells (cellulosic pulp fibers) and plant‐cell‐derived additives in the design of multifunctional thermoplastic paper. a) Schematic presentation of the preparation of plant‐cell‐derived additives. b) Schematic presentation of applying plant‐cell‐derived additives via a surface treatment process that mimics industrial surface sizing used in commercial paper production. c) Digital photograph of plant‐cell‐derived additives. d,e) Demonstration of the scalable preparation process for multifunctional thermoplastic paper.

In this study, we introduce a sustainable, easily scalable concept for transforming conventional paper into a multifunctional material (**Figure** **1**). The paper, characterized by its microstructure composed of cellulosic plant cells formed through standard papermaking processes, undergoes a transformation into thermoplastic paper. This transformation imbues the paper with a range of functional properties, including high wet strength, liquid‐barrier capabilities, antistatic properties, and ductility. These attributes are achieved through the incorporation of green and sustainable additives derived from plant cells. Notably, our concept leverages engineered plant cells as functional additives, enabling the design of paper‐based advanced materials. Our additives generation process involves the creation of hydrodispersible, thermoplastic fibers through in‐situ ring‐opening chemical reactions of fibrils within the porous microstructure of chemically liberated plant cells, followed by dissolution in a non‐derivatizing solvent (Figure 1a). The additives are then integrated into paper's network of plant cells through surface engineering, which involves a surface treatment process (Figure 1b). Through this surface treatment, the plant cells, or cellulosic fibers, within the paper are easily manipulated in situ using sustainable additives. This manipulation includes surface encapsulation, bridging of interfiber gaps, solvent‐induced annealing, and network densification, resulting in a microstructurally reorganized fiber network with a unique combination of functional properties. The multifunctional thermoplastic paper, with its inherent biodegradable nature, is facilely designable, yielding plant‐cell‐derived functional additives and paper‐based products for tailorable end‐use applications at large scale (Figure 1c–e).

## 2 Results and Discussion

### 2.1 From Microstructurally Porous Plant Cells to Cellulosic Additives

**Figure 2:**
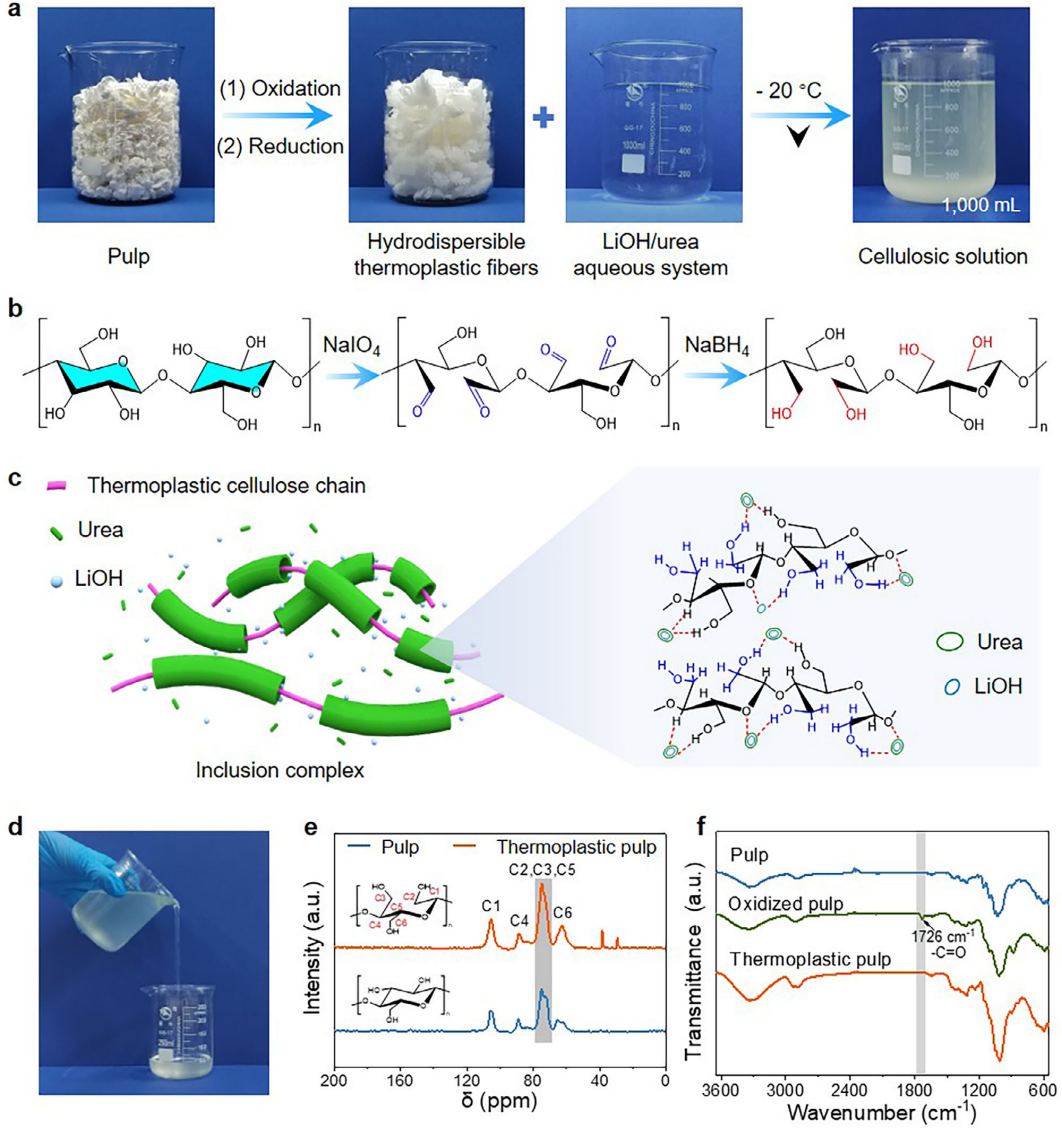
Transformation of liberated plant cells (e.g., pulp fibers) into thermoplastic additives. a) Sequential images illustrating the conversion process of pulp into hydrodispersible, thermoplastic fibers, followed by dissolution in a non‐derivatizing solvent system comprising lithium hydroxide and urea, resulting in the formation of film‐filming cellulosic solutions as additives for paper. b) Schematic presentation of chemical pathways involving ring‐opening of glucose units within cellulosic plant cells. c) Mechanistic depiction of the dissolution process of hydrodispersible thermoplastic fibers within a lithium hydroxide/urea aqueous system. d) Photograph demonstrating the flowability of a cellulosic solution. e) ^13^C solid‐state magic‐angle spinning (MAS) nuclear magnetic resonance (NMR) spectra of untreated and treated pulp fibers. f) Fourier‐transform infrared (FT‐IR) spectra of untreated fiber, NaIO_4_ oxidized fiber and thermoplastic fiber.

Sustainable biorefineries, including those centered on lignocellulose processing,^[^ ^24^, ^25^, ^26^, ^27^ ^]^ hold great promise for the green transformation of materials. Among them, the well‐established pulp and paper industry stands out as one of the most commercially successful examples, offering scalable platforms for valorizing plant‐derived resources into high‐value bioproducts. In this context, our sustainable strategy for designing multifunctional thermoplastic paper is inspired by conventional papermaking approaches, especially surface engineering that conventionally imparts many beneficial properties to paper, including strength, stiffness, and erasability. In this work, we designed thermoplastic plant‐cell‐derived additives to transform regular paper into thermoplastic paper through surface treatment process. Such cellulose‐derived thermoplastic additives are prepared from pulp fibers by ring‐opening modification followed by converting into a cellulosic solution as illustrated in **Figure** **2**a. The raw material for preparing additives is the readily accessible chemical pulp with a highly porous microstructure, which formed due to the removal of lignin and hemicelluloses,^[^ ^28^ ^]^ serving as channels within the microenvironment, facilitating the movement of the chemical reagents, such as sodium periodate and sodium borohydride. The in‐situ ring‐opening reactions of glucose units are described in Figure 2b, the cleavage occurs at the C2‐C3 position of glucosidic rings, this modification decreased the strength of H‐bond network and enhanced the backbone flexibility,^[^ ^29^ ^]^ yielding fibers with enhanced ductility and thermoplasticity.^[^ ^9^, ^30^, ^31^ ^]^ As prepared hydro‐dispersible thermoplastic fibers are characteristic of core‐shell cell wall structure, wherein the shells consist of ring‐opening‐engineered molecules,^[^ ^32^ ^]^ featuring ductile and thermoplastic properties. The hair‐like shells are releasable through mechanical processing,^[^ ^33^, ^34^ ^]^ it can be integrated into the nano‐sized channel of pulp fibers,^[^ ^23^ ^]^ facilitating the formation of sterically stabilized colloids.^[^ ^35^, ^36^, ^37^ ^]^

Non‐derivatizing solvents,^[^ ^38^, ^39^, ^40^, ^41^ ^]^ such as an aqueous mixture of lithium hydroxide and urea, can easily convert the chemically modified lignocellulosic plant cells prepared in this work into cellulosic additives for paper functionalization. When cellulosic materials interact with an aqueous mixture of lithium hydroxide and urea at low temperatures, they form hydrogen‐bond‐induced inclusion complexes,^[^ ^42^ ^]^ wherein Li⁺ and OH− interact with urea molecules, and urea forms hydrogen bonds with both water and cellulose. The complexes formation process disrupts intermolecular hydrogen bonding within the cellulose structure, leading to swelling and solvation, and finally forming a thermoplastic cellulosic chains suspension (Figure 2c). The rheological properties of thermoplastic fiber solution is adjustable (Figures 2d; S1, Supporting Information), which can easily meet the requirements for surface sizing applications on paper products. In nuclear magnetic resonance (NMR) spectra results, as shown in Figure 2e, the increased intensity of the C2 and C3 characteristic peaks of the thermoplastic fibers strongly confirm the success of ring‐opening modification, resulting in structural rearrangement of the cellulose chains. In the Fourier transform infrared (FT‐IR) spectra shown in Figure 2f, the characteristic absorption peak of aldehyde group (─C═O) appears at 1726 cm^−1^ after sodium periodate oxidation, showcasing successful ring‐opening reaction. Due to the instability of the aldehyde group, it is prone to undergo hemiacetal reaction, and aldehyde‐based cellulose is thermosetting. Therefore, it needs to be reduced to hydroxyl groups to stabilize its properties and make it thermoplastic. After sodium borohydride reduction, the characteristic peak of C═O disappears, indicating that aldehyde groups were reduced to hydroxyl groups. Our concept demonstrated herein is dedicated to provide new insights into innovation in multifunctional paper‐based materials by thermoplastic additives derived from lignocellulosic plant cells.

### 2.2 Characterization of the Morphology and Chemical Structure of Thermoplastic Paper

**Figure 3:**
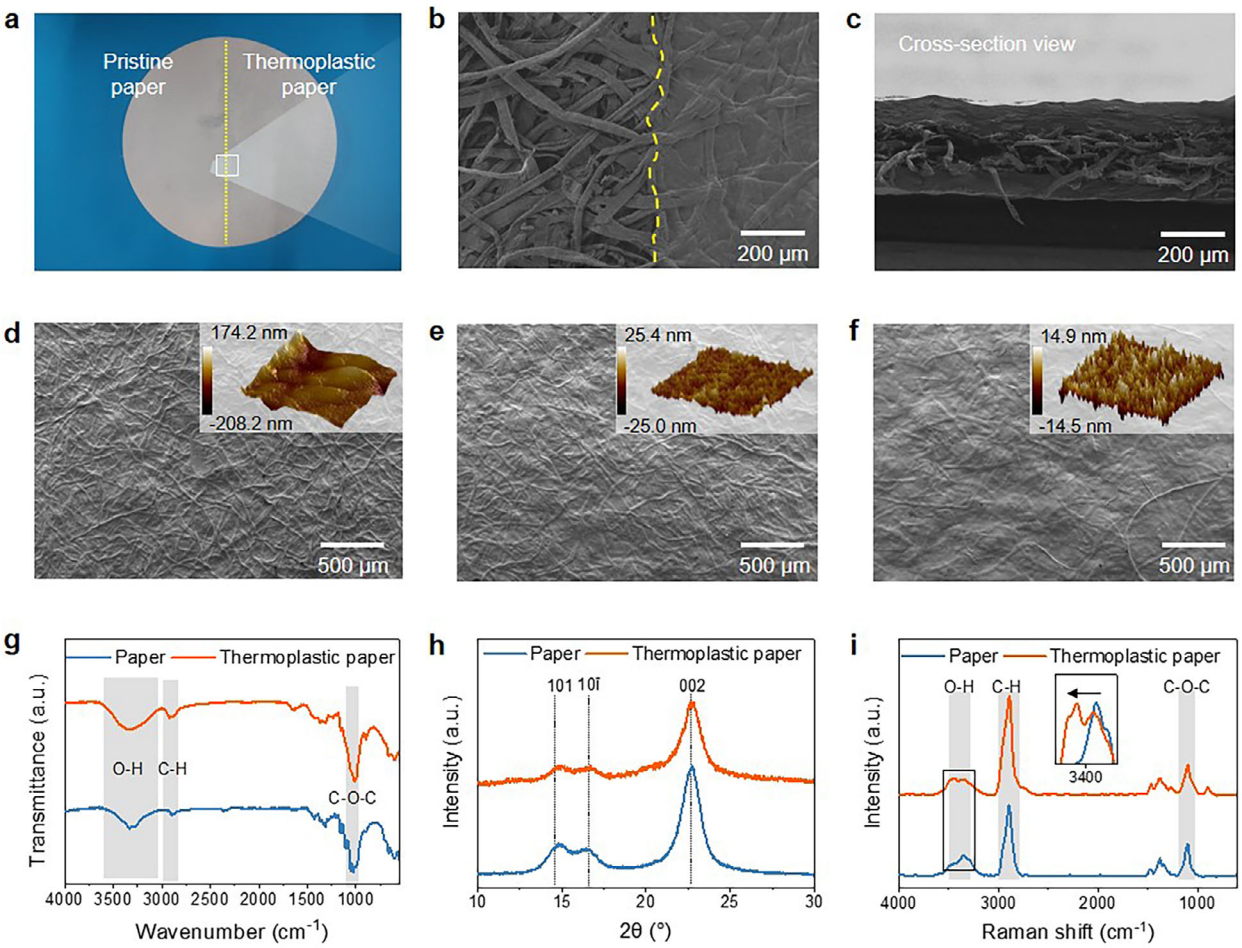
Morphological and chemical characterization. a, b) Top‐view scanning electron micrographs (SEM) illustrating paper morphology before and after a single treatment cycle: A sheet of paper was longitudinally bisected, and micrographs were captured along the midline. c) Cross‐sectional SEM image displaying the internal structure before and after a single treatment cycle. d–f) Top‐view SEM and atomic force microscopy (AFM, 25 µm²) images depicting the evolution of paper morphology after 1, 3, and 5 treatment cycles, respectively. g–i) Fourier‐transform infrared (FTIR) spectra, X‐ray diffraction (XRD) patterns, and Raman spectra of the paper after three treatment cycles.

Plant‐cell‐derived additives can finely tune the surface morphology and topography, as shown in **Figure** **3**a–f. The untreated paper in this work is a typical grade of commercially available cellulosic paper products, which has a porous structure. The thermoplastic additives act as surface coating, percolating into the gaps between fibers and forming a tightly packed structure. As shown by the scanning electron microscope (SEM) images (Figure 3b,c), the surface of paper is filled with additives, but the center of paper is still absent of additives, with regard to this, we conducted several surface sizing treatments to make sure the additives fully impregnate into papers (Figure S2, Supporting Information). SEM images and height‐distance curves obtained from atomic force microscope (AFM) clearly show the smoothening effect of paper treatment with additives (Figure 3d–f). Such a pronounced effect is well known in the case of the surface sizing of paper, which is widely commercially practiced in the paper industry. Generally, surface sizing involves aqueous dispersions of biopolymeric materials such as oxidized starch.^[^ ^43^ ^]^ It is necessary to visualize and qualify the effect of surface sizing using techniques such as atomic force microscopy in terms of printing and many other end‐use applications.^[^ ^44^ ^]^ Indeed, our concept of paper functionalization inherently fits into the commercial unit operations of paper production. The use of plant‐cell‐derived additives can form a transparent coating on cellulosic paper and integrate into the gaps between fibers, resulting in decreased porosity, higher density (Figure S3, Supporting Information), slight size shrinkage (Figure S4, Supporting Information), and increased basis weight (Figure S5, Supporting Information) of the sheet. In the Fourier transform infrared (FT‐IR) spectroscopic result, a broader characteristic band at ≈3330 cm^−1^ (O─H stretching) appears after the additives treatment, indicating an enhanced H‐bond network formed within cellulosic paper^[^ ^45^ ^]^ (Figure 3g). X‐ray diffraction (XRD) results show that thermoplastic paper has lower crystallinity than regular paper (Figure 3h), which is in according with the fact that the thermoplastic additives is amorphous structure with good movability of molecular chains. In Raman spectra (Figure 3i), characteristic peaks of hydroxyl group (─OH) and β1,4‐glycosidic bond (C─O─C) in cellulose are clearly identified at ≈3400 cm^−1^ and 1096 cm^−1^, respectively. The characteristic peak of ‐OH groups exhibited a significant red shift after the addition of additives, indicating a notable change in the hydrogen bonding network within the system.

### 2.3 Mechanical and Thermodynamic Properties of Thermoplastic Paper

**Figure 4:**
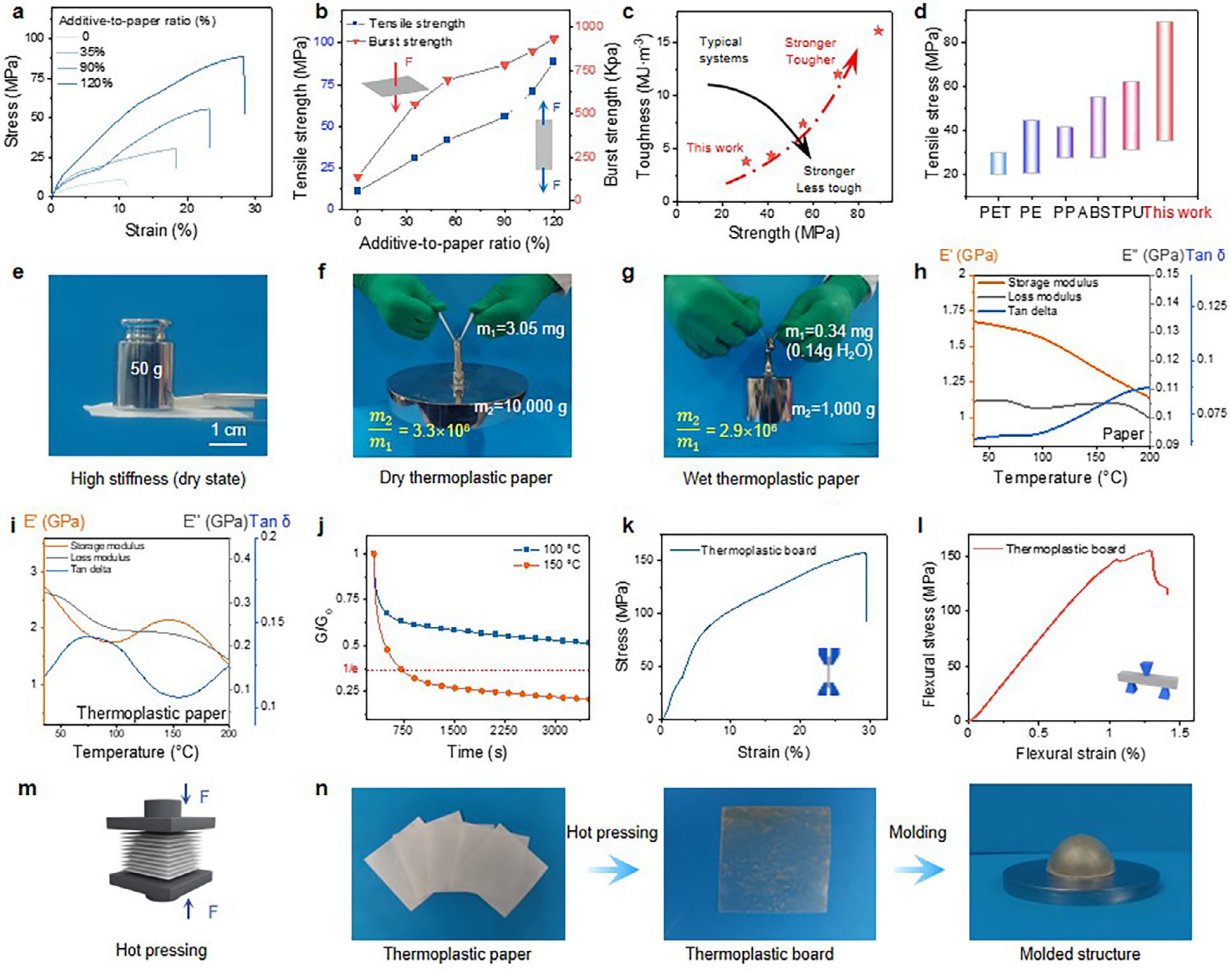
Mechanical and thermoplastic properties. a) Tensile stress–strain curves (dry state) of regular paper and thermoplastic papers with varying additive‐to‐paper weight ratios. b‐d) Strength and toughness comparison showing simultaneous enhancement relative to regular methods and petroleum‐based plastics. e‐g) Photographs of thermoplastic paper exhibiting high stiffness, dry strength, and wet strength. h‐j) DMA and stress relaxation results at 100 °C and 150 °C. k,l) Mechanical performance of thermoplastic board under tension and flexure. n) Hot‐pressing process and thermoplastic formability demonstration.

The inherently low toughness of traditional paper significantly limits its applicability across various fields.^[^ ^46^ ^]^ Interestingly, as shown in this work, the mechanical properties of cellulosic paper, such as tensile and burst strengths, can be significantly enhanced whether in a dry state or under wet conditions (e.g., soaking in water) through the application of plant‐cell‐derived additives. The stress–strain curves clearly show the role of plant‐cell‐derived additives in enhancing paper's strength, ductility, and toughness. Under the conditions studied, increasing the additive‐to‐paper weight ratio can enhance the tensile strength, toughness, and burst strength (**Figures** **4**a,b; S6, Supporting Information). Traditional approaches to improving the mechanical properties of materials often face a trade‐off between strength and toughness, where one property improves at the expense of the other. In contrast, our approach enables the simultaneous enhancement of both strength and toughness in paper (Figure 4c). Figure 4d shows that the tensile strength of the thermoplastic paper exceeds that of standard engineering plastics. Unlike regular paper, the thermoplastic paper exhibits mechanical robustness (Figure 4e). Encouragingly, the thermoplastic paper also exhibits excellent wet strength. In the dry state, a paper strip can support a load equivalent to approximately 3.3 million times its own weight, and even after immersion in water for 20 minutes, it still bears about 2.9 million times its weight. (Figure 4f,g). The elongation at break can increase by up to 40% under wet conditions at room temperature (Figure S7, Supporting Information). The pronounced wet‐strength enhancement is attributed to strengthened interfacial interactions and a densely packed structure, which restrict water molecules from penetrating the paper’s interior.

Moreover, the use of plant‐cell‐derived additives softens the paper, enhancing its tactility and foldability. (Figure S8, Supporting Information). This softening effect is associated with enhanced ductility of fibers within the paper and microscopically smoothened paper surfaces. Notably, the enhanced ductility of fibers is related to the presence of alcohol cellulose, a more flexible macromolecule than native cellulose.^[^ ^47^, ^48^, ^49^, ^50^ ^]^ Similarly, based on the ring‐opening‐based reaction pathways, hemicelluloses such as xylan in cellulosic fibers are convertible into their alcohol forms.^[^ ^51^ ^]^ The additives used in this work for paper treatment contain dial‐alcohol‐based structures that facilitate the softening of fibers.

Due to the extensive hydrogen bonding networks both within and between cellulose chains, native cellulose is inherently stable and resistant to melting or softening upon heating, instead, it undergoes irreversible chemical changes, resulting in decomposition or char.^[^ ^52^ ^]^ In our work, the ring‐opening modification disrupts the hydrogen‐bonding networks and imparts mobility to the molecular chains, thereby rendering the additives thermoplastic. The enhanced ductility of fibers is related to the presence of dialcohol cellulose, a more flexible macromolecule than native cellulose.^[^ ^47^, ^48^, ^49^, ^50^ ^]^ Similarly, built upon the ring‐opening‐based reaction pathways, hemicelluloses such as xylan in cellulosic fibers are also convertible into their dialcohol forms. Dynamic mechanical analysis (DMA) was performed to investigate the heat‐induced malleability of the thermoplastic paper. The time‐ and temperature‐dependent stress relaxation behavior of the thermoplastic paper was also examined at elevated temperatures (100 and 150 °C) using DMA (Figure 4h). The time for relaxation of 1/e stress is defined as the relaxation time (τ). Similar to other thermoplastics, the τ of thermoplastic paper at 150 °C was 12.5 min. The storage modulus of regular paper (Figure 4i) constantly decreases as the increase of temperature, but the storage modulus of thermoplastic paper (Figure 4j) exhibits a peak at 147.79 °C, implying that the mobility of the modified‐cellulose chain can be improved at high temperatures. Tg of our thermoplastic paper appears at 73.91 °C according to the tan delta curve, which is similar to bio‐based plastics such as PLA (60 °C). Furthermore, the thermoplastic paper was analyzed for temperature‐dependent mechanical performance at 100 and 150 °C using dynamic mechanical analysis (DMA) in tensile mode (Figure S9, Supporting Information). Results from both tensile tests indicate that, with increasing temperature, the elongation at break increases by 90%, while the tensile strength slightly decreases by 25%. These results clearly demonstrate that, after treatment with plant‐cell‐derived additives, regular paper can be transformed into thermoplastic paper. Owing to the thermoplastic property, as depicted in Figure S10 (Supporting Information), the paper treated with plant‐cell‐derived additives can be molded into complex shapes such as waves, spirals, and cylinders. Moreover, multiple thermoplastic papers can be hot‐pressed into transparent thermoplastic boards (Figure 4m), and these boards can then be molded into customizable structures (Figure 4n). The tensile stress‐strain curve of thermoplastic boards is shown in Figure 4k. Its tensile strength reached 157.81 MPa, which is higher than that of most engineering plastics.A three‐point bending test was also conducted on the thermoplastic boards(Figure 4l). The bending strength reaches 154.70 MPa, which is higher than that of polycarbonate (PC) or polyoxymethylene (POM).

### 2.4 Comprehensive Stability of Thermoplastic Paper

**Figure 5:**
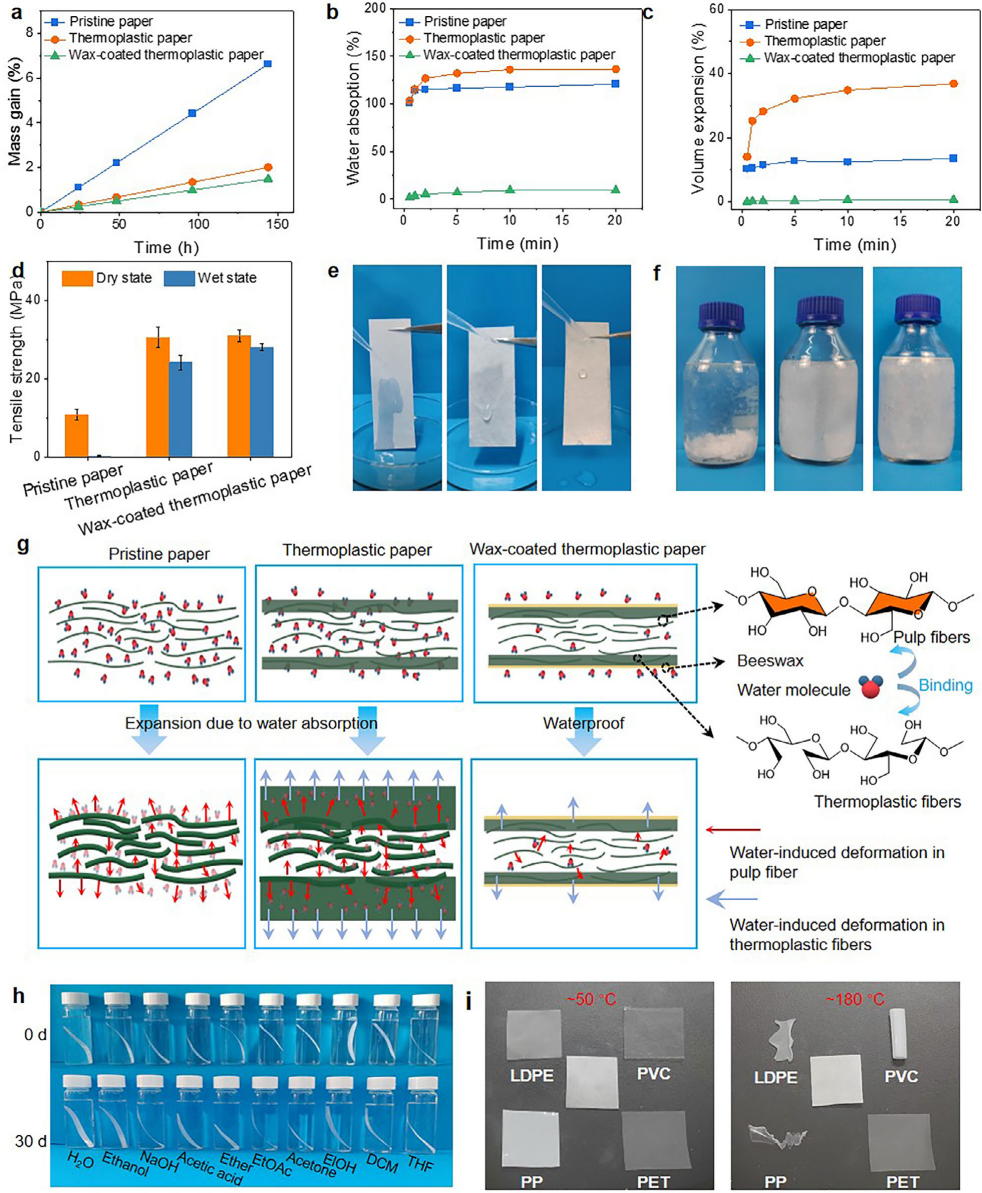
Comprehensive stability of the thermoplastic paper. a) Water uptake of CaCl_2_ in bottles covered by regular paper, thermoplastic paper, and beeswax‐treated thermoplastic paper during 144 h. b) Water absorption after immersion in deionized water for 20 min. c) Volume expansion rate after immersion in deionized water for 20 min. d) Tensile strength before and after immersion in deionized water. g) Schematic illustration of the volume expansion mechanism for paper, thermoplastic paper, and beeswax‐treated thermoplastic paper.h) Solvent stability in deionized water, anhydrous ethanol, NaOH solution, acetic acid, ether, ethyl acetate, acetone, isobutane, dichloroethane, and tetrahydrofuran. i) Thermal stability of thermoplastic paper compared with low‐density polyethylene (LDPE), polyvinyl chloride (PVC), polypropylene (PP), and polyethylene terephthalate (PET).

Owing to the importance of structural stability in practical applications, the water, solvent, and thermal stability of the thermoplastic paper were evaluated. Compared with regular paper, the thermoplastic paper exhibits a diminished water vapor transmission rate, which is attributed to its more homogeneous and denser structure (**Figures** **5**a; S11, S12, Supporting Information). The molecular structure of the additives comprises alcohol‐based chains, which are hydrophilic and soften the fibers while improving their water‐retention capacity ^[^ ^53^, ^54^, ^55^ ^]^. Consequently, the thermoplastic paper exhibits greater water absorption and volume expansion than the base paper. Enhanced hygroscopicity benefits the anti‐static performance, as shown in Figures S13, and S14 (Supporting Information). The role of the additives in reducing the buildup of static electricity (e.g., caused by contact electrification) is associated with their ability to attract water molecules, which dissipate the electrostatic charge^[^ ^56^ ^]^ and dissolve ions that contribute to conductivity.^[^ ^57^, ^58^ ^]^

To improve the water resistance and broaden the potential for plastic substitutiont, an additional surface treatment was applied to the thermoplastic paper. Specifically, the thermoplastic paper was immersed in a beeswax–ethanol solution (5 mL beeswax per 100 mL ethanol) thoroughly mixed at 80 °C for 5 min, followed by drying at 100 °C for 10 min. The treated thermoplastic paper exhibited a markedly reduced water absorption rate of approximately 9.3%, compared with 119.7% for the base thermoplastic paper and 135.3% for the untreated paper. The water absorption profile further demonstrated negligible dimensional change during the test, confirming excellent water stability after hydrophobic treatment (Figure 5b,c). Additionally, both treated and untreated thermoplastic papers maintained good wet strength (Figure 5d). As shown in Figure 4e, water droplets on the left side of the filter paper permeated rapidly, while those on the middle (thermoplastic paper) adhered to the surface without penetration. In contrast, water droplets on the right side (hydrophobic thermoplastic paper) rolled off easily, forming a nearly spherical shape. Regular paper lacked this dissociation resistance, whereas both thermoplastic and hydrophobic thermoplastic papers retained it (Figure 4f). Figure 5g. Due to their abundant hydrophilic groups (─OH), both lignocellulose‐derived pulp fibers and thermoplastic fibers are prone to absorb water^[^ ^24^, ^59^, ^60^ ^]^; however, after coating with beeswax, water penetration can be effectively inhibited.

To further evaluate solvent stability, the thermoplastic paper was immersed in various solvents, including deionized water, anhydrous ethanol, NaOH solution, acetic acid, ether, ethyl acetate, acetone, isobutane, dichloroethane, and tetrahydrofuran (Figure 5h). After 30 days, the thermoplastic paper remained intact in all solvents, showcasing excellent solvent stability. The combination of acceptable solvent resistance and liquid barrier performance indicates strong potential for packaging applications (Figure S15, Supporting Information).

Thermal stability of the thermoplastic paper was also tested, as shown in Figure 5i. The thermoplastic paper shows no visible change below 180 °C, whereas low‐density polyethylene (LDPE), polyvinyl chloride (PVC), and polypropylene (PP) begin to melt to varying extents. Thermogravimetric analysis (TGA) results are presented in Figure S16 (Supporting Information). The thermoplastic paper remains stable up to 250–300 °C, and the onset of weight loss corresponds to thermal decomposition of cellulose.

### 2.5 Recyclability and Degradability of Thermoplastic Paper

**Figure 6:**
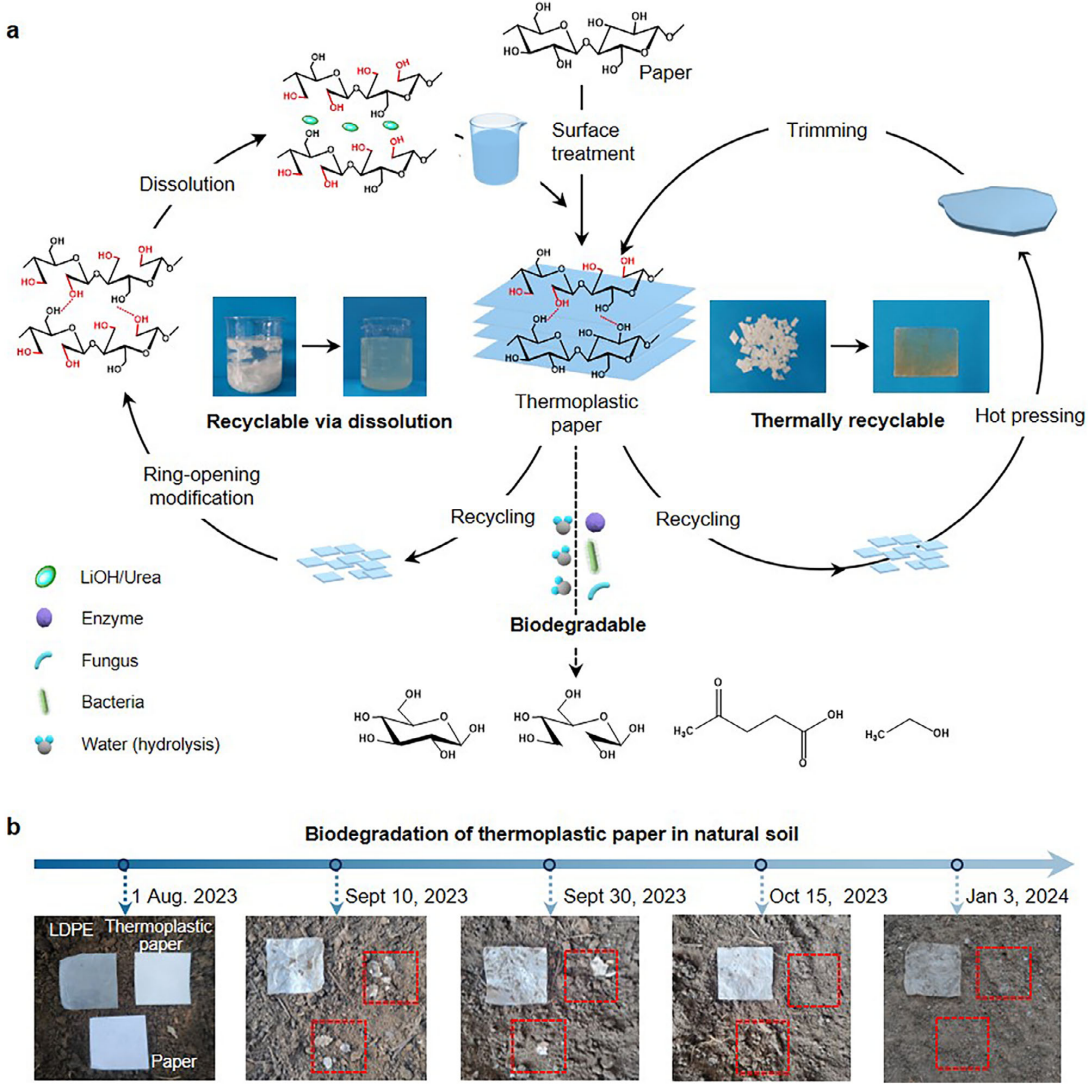
Environmental impact of thermoplastic paper. a) Closed‐loop life‐cycle scheme illustrating the dissolution‐induced recyclability, thermal reprocessability, and biodegradability of thermoplastic paper. b) Soil burial test comparing the degradation behavior of low density polyethylene (LDPE), regular paper, and thermoplastic paper under natural soil conditions.

Excellent recyclability and degradability are pivotal in plastic replacement.^[^ ^61^ ^]^ As depicted in **Figure** **6**a, thermoplastic paper prepared through surface treatment with plant‐cell‐derived additives can be recycled via dissolution. The newly obtained additives can be used to treat another regular paper. On the other hand, thermoplastic paper can be thermally recycled: The used products can be re‐shaped into new products through simple slicing and hot‐pressing process. This procedure establishes a closed‐loop recycling system and re‐utilization of thermoplastic paper, facilitating efficient resource recovery and regeneration. Figures S17 and S18 (Supporting Information) demonstrate the effect of number of recycling cycles on the mechanical properties. Due to the degradation of cellulose chain, the mechanical properties of thermoplastic paper can slightly decrease after each cycle.

The biodegradability of thermoplastic paper was further evaluated, as shown in Figure 6b. With burying in natural soil, both thermoplastic and regular paper can be decomposed into fragments within two months, and fully decomposed after five months, suggesting their excellent biodegradability and good environmental friendliness. As for comparison, low‐density polyethylene (LDPE) shows poor biodegradability. Of note, the balance between stability and biodegradability has been a long‐standing challenge for biodegradable products. Our thermoplastic paper can balance these two properties very well, as the thermoplastic paper is super stable in their daily use environment which is away from microorganisms (e.g., bacteria and fungi), whereas easily degradable when disposed after its end of life. The manifestation of biodegradability and recyclability suggests that thermoplastic paper will have a lesser environmental impact than conventional plastics, which represents a promising candidate of sustainable alternatives for petro‐based plastic substitution.

## 3 Conclusion

In the context of ever‐increasing demand for sustainable materials for replacements of non‐renewables or difficult‐to‐degrade plastics, we herein present a facile concept of designing cellulosic multifunctional thermoplastic paper based on plant cells (pulp fibers) and plant‐cell‐derived additives. Such additives are easily formable due to ring‐opening‐based heterogeneous chemical modification of pulp followed by dissolving in an aqueous medium, which generates cellulosic additives for paper treatment. Essentially, the chemical processing of pulp based on the ring‐opening of glucosidic rings leads to increased flexibility and ductility of some biomacromolecules of fibers, which can be formed into a cellulosic solution containing various forms of dissolved/disassembled organics. Using plant‐cell‐derived additives can deliver thermoplastic, ductile, wet‐strength, oxygen‐barrier, liquid‐barrier, and antistatic properties to paper, which can be regarded as a facile strategy of paper‐based “modern alchemy”. The engineered paper is hot‐pressable and 3D‐formable, which allows easy applicability to diversified uses and applications. Other advantages of the concept involve adaptability to papermaking unit operations associated with surface engineering (e.g., surface sizing) of paper, designability of paper structure, and performance based on different cycles of treatments, the possibility of forming advanced products based on lamination.

## Conflict of Interest

The authors declare no conflict of interest.

## Supporting information



Supporting Information

Supplementary Zip

Supplementary Zip

Supplementary Information

## Data Availability

The data that support the findings of this study are available in the supplementary material of this article.
