# Supplementary material for: Multifunctional Thermoplastic Paper Enabled by Plant‐Cell‐Derived Additives: A Paradigm of Paper‐Based “Modern Alchemy”
Source: Adv Sci (Weinh). 2025 Nov 5;13(2):e06157. doi: 10.1002/advs.202506157 (PMC12786277; doi:10.1002/advs.202506157)
Supplement: Supplementary file 1 — Supporting Information [file ADVS-13-e06157-s003.docx]

**Supplemental Information: Experimental Section and Figures**

Multifunctional Thermoplastic Paper Enabled by Plant-Cell-Derived Additives: A Paradigm of Paper-Based “Modern Alchemy”

Xiaoyan Yu^1,2,3‡^, Jie Zhou^2,‡^, Jianqiang Li^4^, Hongyang Yuan^1^, Xueren Qian^1^, Yonghao Ni^5,6^, Zhibin He^5^, Chaoji Chen^2,^*, Jing Shen^1,5,^*

*^1^ Research Division for Sustainable Papermaking & Advanced Materials, Key Laboratory of Biobased Materials Science and Technology (Ministry of Education), Northeast Forestry University, 26 Hexing Road, Harbin 150040, China.*

*^2^ School of Resource and Environmental Sciences, Hubei Biomass-Resource Chemistry and Environmental Biotechnology Key Laboratory, Wuhan University, Wuhan, 430079 China.*

*^3^ Engineering Research Center for Hemp and Product in Cold Region of Ministry of Education, School of Light Industry and Textile, Qiqihar University, Qiqihar 161006, China.*

*^4^ Shandong Huatai paper Co. Ltd., Dongying, 257335, China.*

*^5^ Limerick Pulp and Paper Centre, Department of Chemical Engineering, University of New Brunswick, Fredericton, NB E3B 6C2, Canada.*

*^6^ Department of Chemical and Biomedical Engineering, University of Maine, Orono, ME 04469, United States.*

* Email: chenchaojili@whu.edu.cn (C.C.); jingshen.china@hotmail.com (J.S.)

^‡^These authors contributed equally to this work.

**Experimental**

**Materials.** Bleached softwood kraft pulp (liberated plant cells) imported from Russia was supplied by Liaoning Jiali Trade Co., Ltd., China. Quantitative filter paper, a semi-permeable material with medium filtration speed, was obtained from Hangzhou Specialty Paper Co., Ltd., China. Sodium periodate (99.5%), sodium borohydride (97%), lithium hydroxide (analytical reagent grade), urea (analytical reagent grade), and anhydrous ethanol (analytical reagent grade) were purchased from Tianjin Kermio Chemical Reagents Co., Ltd., Tianjin Tianli Chemical Reagents Co., Ltd., Shanghai Maikelin Biochemical Technologies Co., Ltd., and Shanghai Chemical Reagents Co., Ltd., respectively. Deionized water was used throughout the study.

**Preparation of Plant-Cell-Derived Additives.** The mechanically refined wet pulp, with an oven-dry mass of 20 g, was added to a three-neck round-bottom flask containing 1,000 mL of water. The pulp and water were thoroughly mixed using an overhead mechanical stirrer. Sodium periodate was then added, and the oxidation reaction was carried out for 2 h at 50 °C in a water bath under stirring. The reaction was quenched by washing the pulp sufficiently to remove unreacted sodium periodate. Subsequently, the reduction reaction was performed for 6 h using sodium borohydride at a concentration of 2.5 g/L (pulp consistency: 4 g/L). This reaction was also quenched by thorough pulp washing. Aqueous plant-cell-derived additives (8 wt%) were prepared by dispersing thermoplastic fibers (8 g, oven-dry basis) into a precooled (−20 °C) aqueous solution (92 g, containing 4.6 wt% LiOH, 15 wt% urea, and 72.4 wt% H₂O).

**Preparation of Thermoplastic Paper.** The aqueous plant-cell-derived additives containing dissolved cellulosic materials with ring-opened structures, together with other components (e.g., urea), were used to treat quantitative filter paper by bar coating. Each coating cycle produced a liquid film with a thickness of 100 μm on both sides. After coating, the samples were immersed in anhydrous ethanol for 10 min and repeatedly rinsed with water. The samples were then air-dried naturally. In some cases, the dried samples were further processed at 150 °C for 5 min under 30 MPa using an R32022015 hot-press machine (China).

**Physicochemical and Morphological Characterizations.** Fourier-transform infrared (FTIR) spectroscopy, X-ray diffraction (XRD), morphological, topographical, Raman spectroscopy, solid-state ^13C nuclear magnetic resonance (^13C NMR), and thermogravimetric analyses were conducted using the following instruments: a Nicolet Magna-IR 560 spectrometer (Thermo Fisher Scientific, United States), a PANalytical X’Pert PRO X-ray diffractometer (Malvern PANalytical, Netherlands), a Zeiss Supra 55 scanning electron microscope (Carl Zeiss, Germany), a Bruker Dimension Icon atomic force microscope (Bruker, Germany), a LabRAM Xplora confocal Raman microscope (Horiba, France), a JEOL JNM-ECZ600R solid-state NMR spectrometer (JEOL, Japan), and a TA Instruments thermogravimetric analyzer (TA Instruments, United States).

**Evaluation of Water Vapor Transmission, Water Absorption, Solvent Resistance, and Barrier Performance.** Water vapor barrier properties were determined by measuring the weight change of calcium chloride (CaCl₂) in a sealed bottle over 144 h under an air atmosphere with saturated salt water at 25 °C. The water vapor transmission rate (WVTR) was calculated from the ratio of the mass change of CaCl₂ to the effective area per day (expressed in g m⁻² d⁻¹). Water absorption was evaluated based on the weight change of regular paper, thermoplastic paper, and treated thermoplastic paper before and after immersion in deionized water for 20 min. The shape stability was demonstrated by visually recording the drying, wetting, and redrying states of the samples. Solvent resistance was assessed by immersing thermoplastic paper in various solvents—deionized water, anhydrous ethanol, NaOH, acetic acid, ether, ethyl acetate, acetone, isobutane, dichloroethane, and tetrahydrofuran—for 30 days. Oxygen transmission rates were measured using a BTY-B3P gas permeability tester (China) at 20% relative humidity. In addition, glass filters were employed to evaluate the barrier performance against deionized water, plant-based cooking oil, and milk under gravity.

**Evaluation of Structural, Mechanical, and Thermal Properties.** Paper density was calculated based on data obtained using a ZUS-4 paper thickness tester (China). Paper porosity was determined by soaking the samples in benzene for 8 h, removing unabsorbed excess liquid, and measuring the mass change, followed by calculations using the ratio of mass change to the product of liquid density and sheet volume. The softness of the paper was measured with an AT-RR softness tester (China). Mechanical properties were evaluated using a CMT6103 universal mechanical strength tester (MTS Systems, United States) and a BSM-1600 burst strength tester (China). For wet-strength tests, the samples were immersed in deionized water for 20 min and surface moisture was gently removed prior to testing. The temperature-dependent tensile behavior of thermoplastic paper was characterized using a dynamic mechanical analyzer (DMA Q800, TA Instruments, United States). In addition, the DMA and stress relaxation analyses of thermoplastic paper were performed with a Q850 DMA instrument (TA Instruments, United States). Thermal stability was examined using a thermogravimetric analyzer (TA Instruments, United States) from 30 °C to 600 °C at a heating rate of 20 °C min⁻¹ under a nitrogen atmosphere.

**Biodegradability Testing.** Original paper, thermoplastic paper, and low-density polyethylene (LDPE) film (each cut to 50 mm × 50 mm) were buried in soil at a depth of 5 cm under ambient outdoor conditions. The samples were retrieved at predetermined time intervals for visual observation and biodegradation assessment.


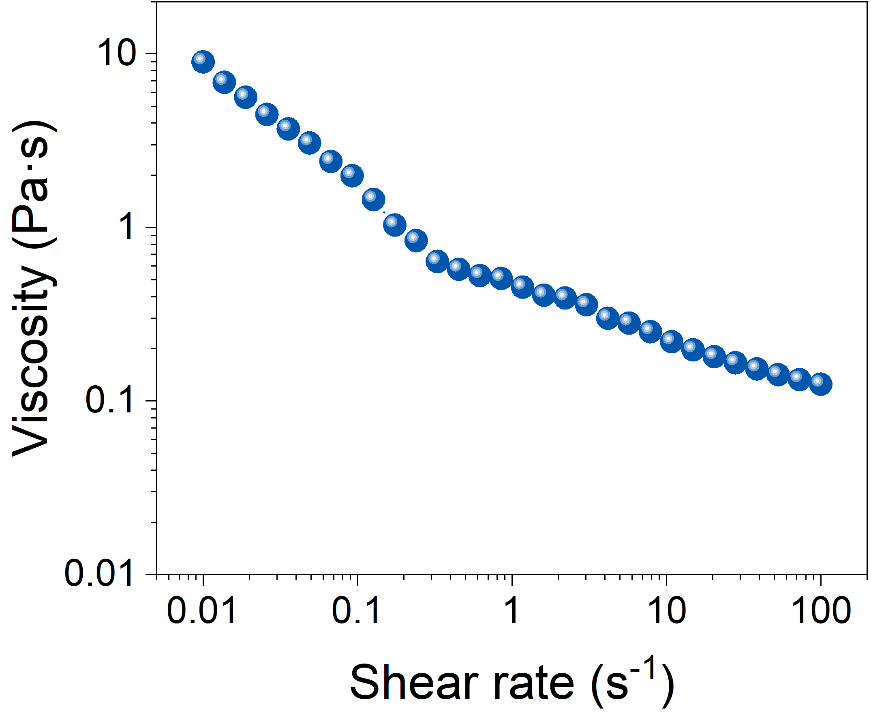


**Figure S1**. Viscosity–shear rate relationship of the cellulosic solution measured at room temperature. The solution exhibits typical non-Newtonian shear-thinning behavior, characterized by a progressive decrease in viscosity with increasing shear rate. This behavior reflects the alignment and orientation of polymer chains under applied shear stress, which is characteristic of cellulose-based systems.


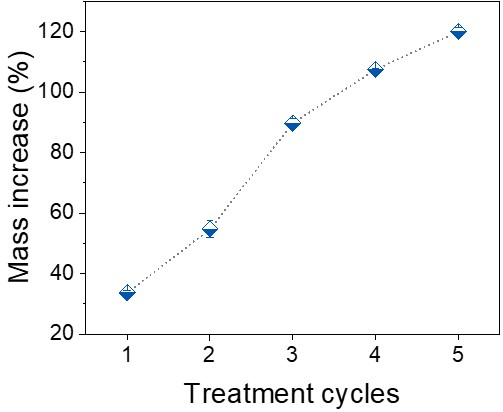


**Figure S2.** Mass ratio (%) of thermoplastic paper as a function of the number of surface treatment cycles. The mass ratio was determined gravimetrically according to Equation (1).

$C \left( wt \% \right)=\frac{m_{2}-m_{1}}{m_{1}}\times100\%$ (1)

where m₁ is the oven-dry mass of the regular paper, and m₂ is the oven-dry mass of the paper after thermoplastic treatment.


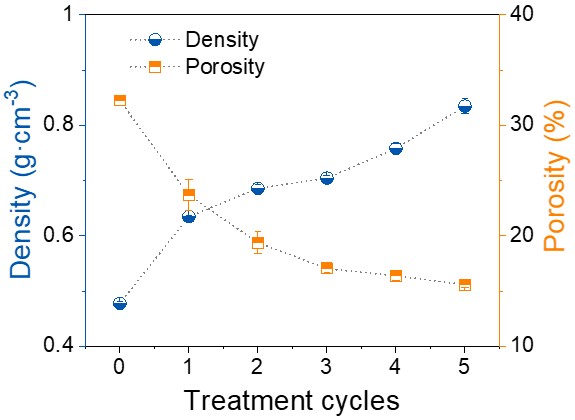


**Figure S3.** Evolution of density and porosity of thermoplastic paper as a function of the number of surface treatment cycles. The untreated paper (0 cycle) is included as a reference. Density increases, while porosity decreases, with an increasing number of additive treatments.


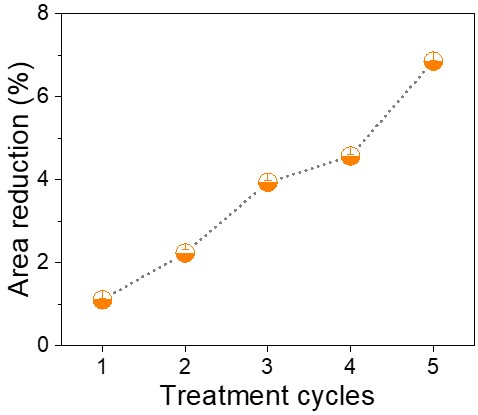


**Figure S4.** Area reduction (%) of thermoplastic paper as a function of the number of surface treatment cycles. Increased treatment leads to greater shrinkage during hot-pressing, indicating progressive structural densification.


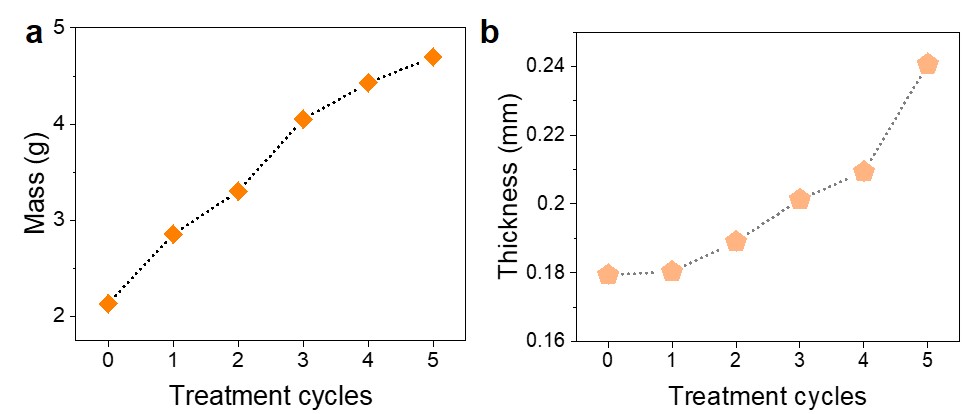


**Figure S5.** Effect of the number of surface treatment cycles on the (a) mass and (b) thickness of thermoplastic paper. Both parameters increase progressively with successive treatments, indicating additive accumulation.


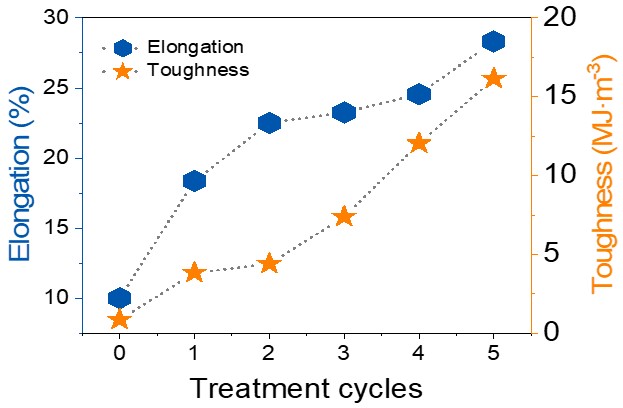


**Figure S6.** Effect of the number of surface treatment cycles on the elongation and toughness of thermoplastic paper under dry conditions. Both properties increase progressively with successive additive applications, indicating enhanced ductility and improved energy-absorption capacity.


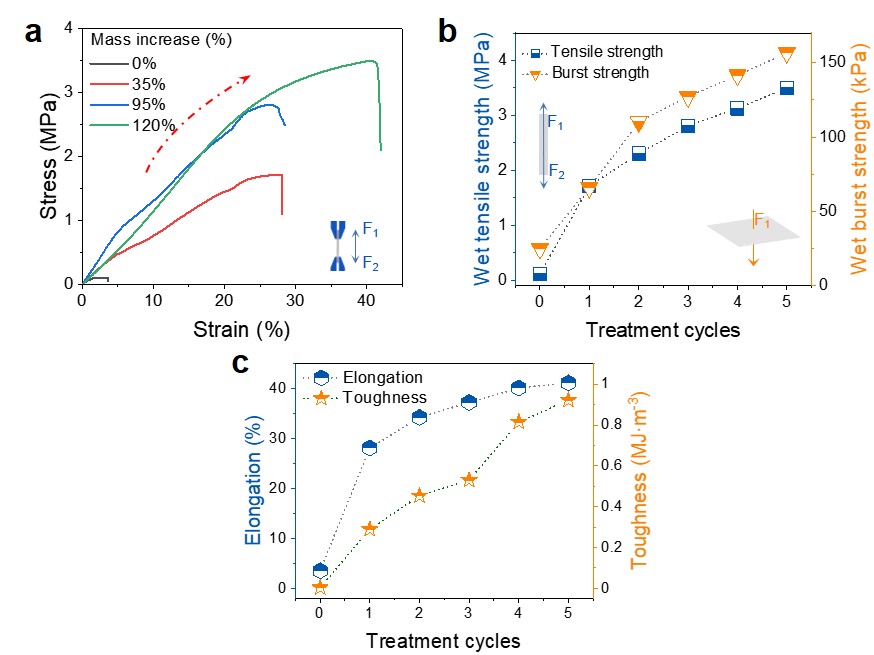


**Figure S7.** Mechanical properties of thermoplastic paper after soaking in water for 20 min. (a) Tensile stress–strain curves of regular paper and thermoplastic papers with different mass increases. (b) Variation in wet tensile strength and burst strength with the number of surface treatment cycles. (c) Enhancement of elongation and toughness relative to untreated paper, highlighting the strengthening and toughening effects of the thermoplastic additive strategy. F₁ and F₂ indicate characteristic stress points on the stress–strain curves.


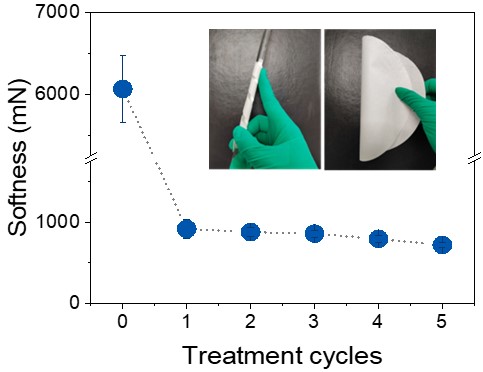


**Figure S8.** Effect of the number of surface treatment cycles on the softness of thermoplastic paper. The significant decrease in measured force indicates improved material softness with increasing treatment. Softness was measured using an AT-RR softness tester (China). Samples were conditioned at 75% relative humidity for 48 h prior to testing.

**Figure S9.** Thermomechanical behavior of thermoplastic paper at elevated temperatures. (a) Stress-relaxation curves at 100 °C and 150 °C showing time-dependent decay of modulus under constant strain. Higher temperatures lead to faster relaxation and lower modulus values. (b) High-temperature tensile stress–strain curves demonstrating reduced tensile strength and enhanced ductility at 150 °C compared with 100 °C.

**Figure S10.** Shape-morphing ability and mechanical robustness of thermoplastic paper. Starting from flat sheets (left column), the paper can be readily molded into spiral, wave, and cylindrical shapes (middle column). These formed structures exhibit excellent load-bearing capability, supporting weights from 50 g to 1,000 g without noticeable deformation (right column).

**Figure S11** Experimental setup for measuring water vapor transmission rate (WVTR). (a) Bottles containing anhydrous CaCl₂ were used to absorb moisture permeating through the test samples. (b) The test was conducted in a sealed desiccator with controlled humidity maintained by a saturated NaCl solution to simulate a stable humidity environment.


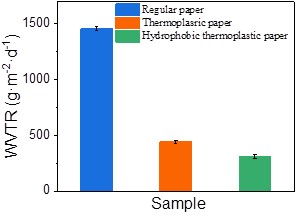


**Figure S12.** Water vapor transmission rate (WVTR) of regular paper, thermoplastic paper, and hydrophobic thermoplastic paper. The WVTR decreases markedly after additive treatment and is further reduced by hydrophobic modification, indicating enhanced moisture-barrier performance.


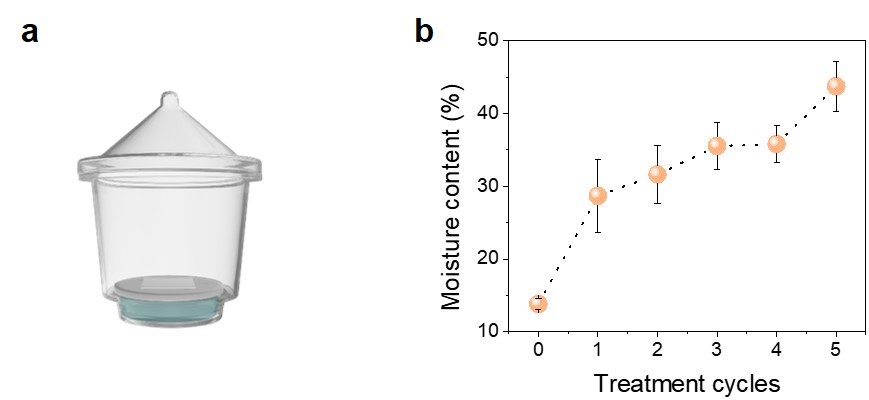


**Figure S13.** Moisture-absorption behavior of thermoplastic paper under high-humidity conditions. (a) Schematic of the experimental setup employing a sealed chamber containing a saturated NaCl solution to maintain 75 % RH. (b) Moisture content of thermoplastic paper after equilibrium exposure at 75 % RH for 48 h. Moisture uptake increases with additive treatment cycles, indicating enhanced hygroscopicity.


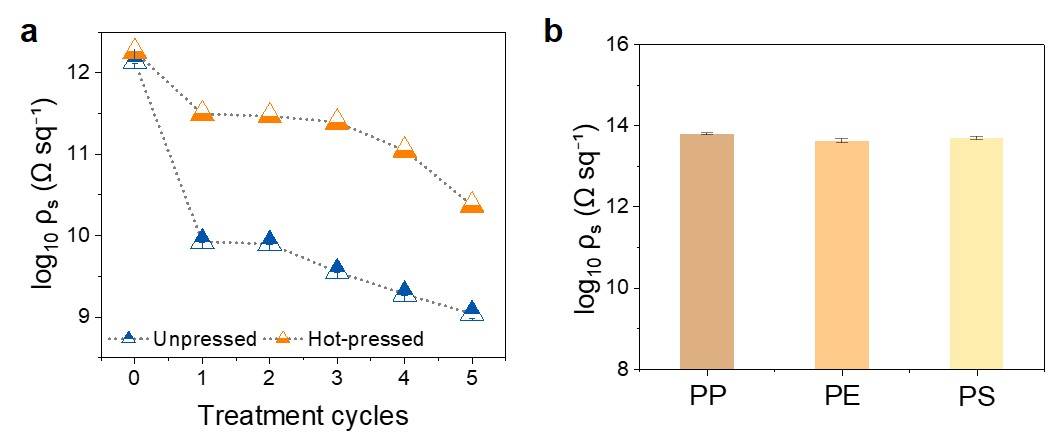


**Figure S14**. Antistatic properties of thermoplastic paper compared with common plastics. (a) Surface resistivity (log₁₀ scale) of thermoplastic paper with different treatment cycles under unpressed and hot-pressed conditions. (b) Surface resistivity of typical plastic materials, including polypropylene (PP), polyethylene (PE), and polystyrene (PS). With increasing treatment cycles, thermoplastic paper—particularly after hot pressing—exhibits markedly reduced surface resistivity, approaching or surpassing the antistatic threshold (log₁₀ ρₛ ≈ 9), indicating improved antistatic performance.


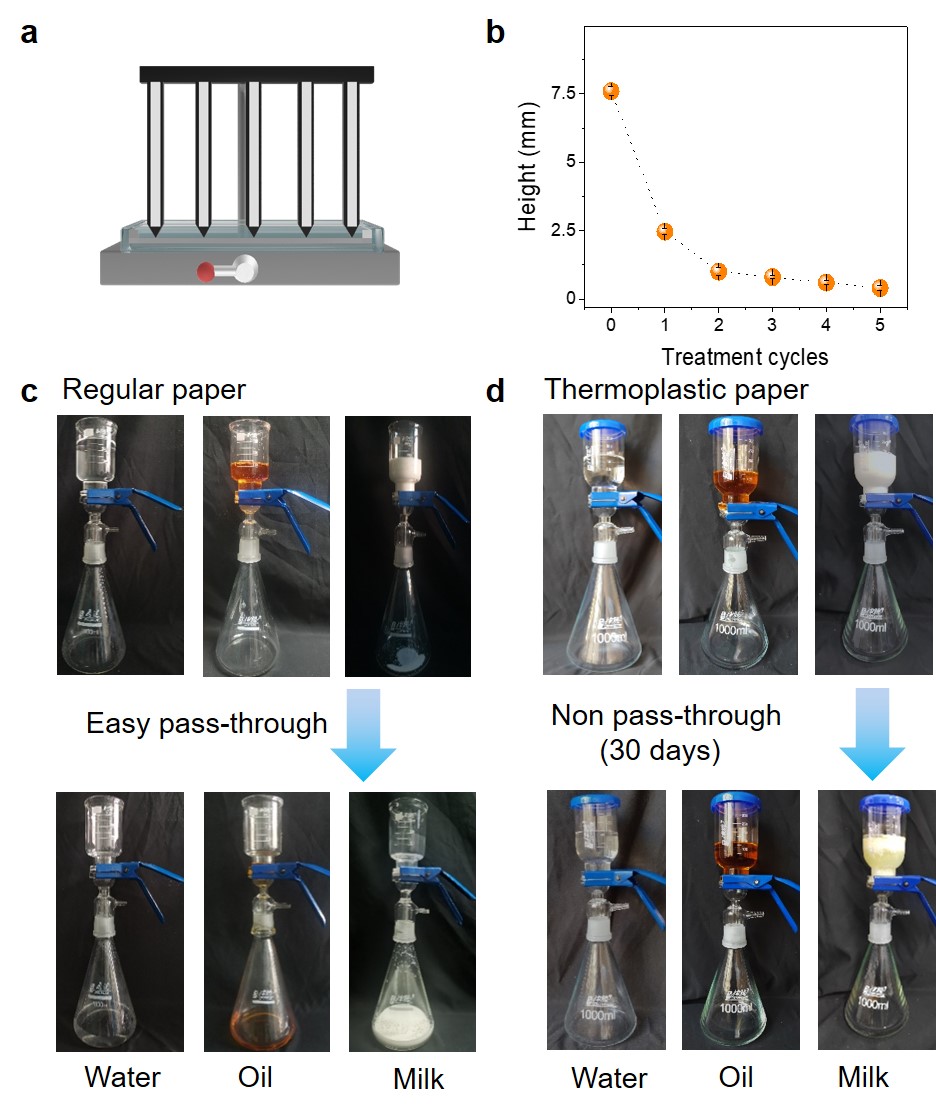


**Figure S15.** Effect of additive treatment on capillary rise behavior and liquid barrier performance of paper. (a) Schematic of the capillary suction test apparatus used to evaluate paper wetting behavior. (b) Capillary rise height as a function of treatment cycles, showing a pronounced reduction with increased additive application. (c) Photographs showing rapid permeation of water, oil, and milk through untreated paper. (d) Photographs showing that thermoplastic paper effectively blocks the penetration of water, oil, and milk even after 30 days.


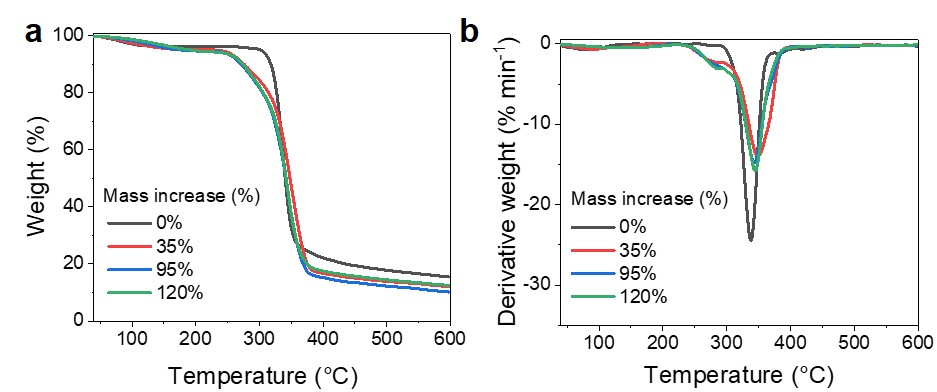


**Figure S16.** Thermogravimetric analysis (TGA) of thermoplastic paper with varying degrees of mass increase. (a) TG curves showing weight loss (%) as a function of temperature. (b) DTG curves (derivative thermogravimetry) illustrating the decomposition rate. The introduction of thermoplastic material slightly lowers the onset degradation temperature but maintains overall thermal stability, indicating that the additive does not markedly compromise the paper’s thermal resistance.


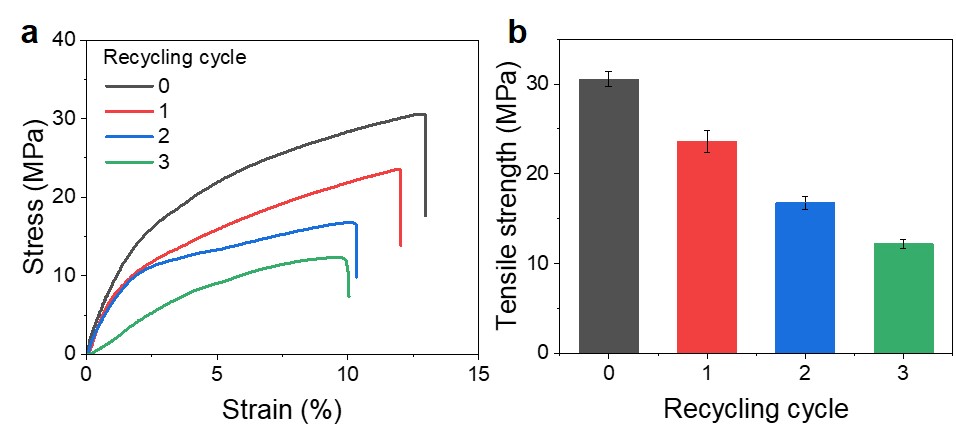


**Figure S17.** Effect of recycling cycles on the tensile performance of thermoplastic paper. (a) Tensile stress–strain curves of samples subjected to 0–3 dissolution–regeneration cycles. (b) Corresponding tensile strength values showing gradual mechanical degradation with increasing recycling. The decrease in strength and elongation suggests partial deterioration of the thermoplastic network and structural reorganization during repeated dissolution.


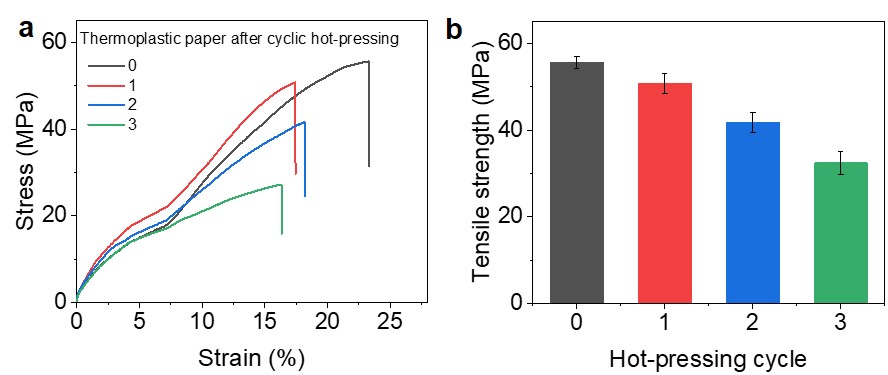


**Figure S18.** Effect of repeated hot-pressing cycles on the mechanical properties of thermoplastic paper. (a) Tensile stress–strain curves of samples subjected to 0–3 hot-pressing cycles. (b) Corresponding tensile strength values after each reshaping cycle. Repeated thermal reshaping results in a gradual decrease in both tensile strength and elongation, likely caused by structural fatigue, chain relaxation, or partial densification of the regenerated matrix.
