# Supplementary figures and images for: Multifunctional Thermoplastic Paper Enabled by Plant‐Cell‐Derived Additives: A Paradigm of Paper‐Based “Modern Alchemy”
Source: Adv Sci (Weinh). 2025 Nov 5;13(2):e06157. doi: 10.1002/advs.202506157 (PMC12786277; doi:10.1002/advs.202506157)

## Slide 1
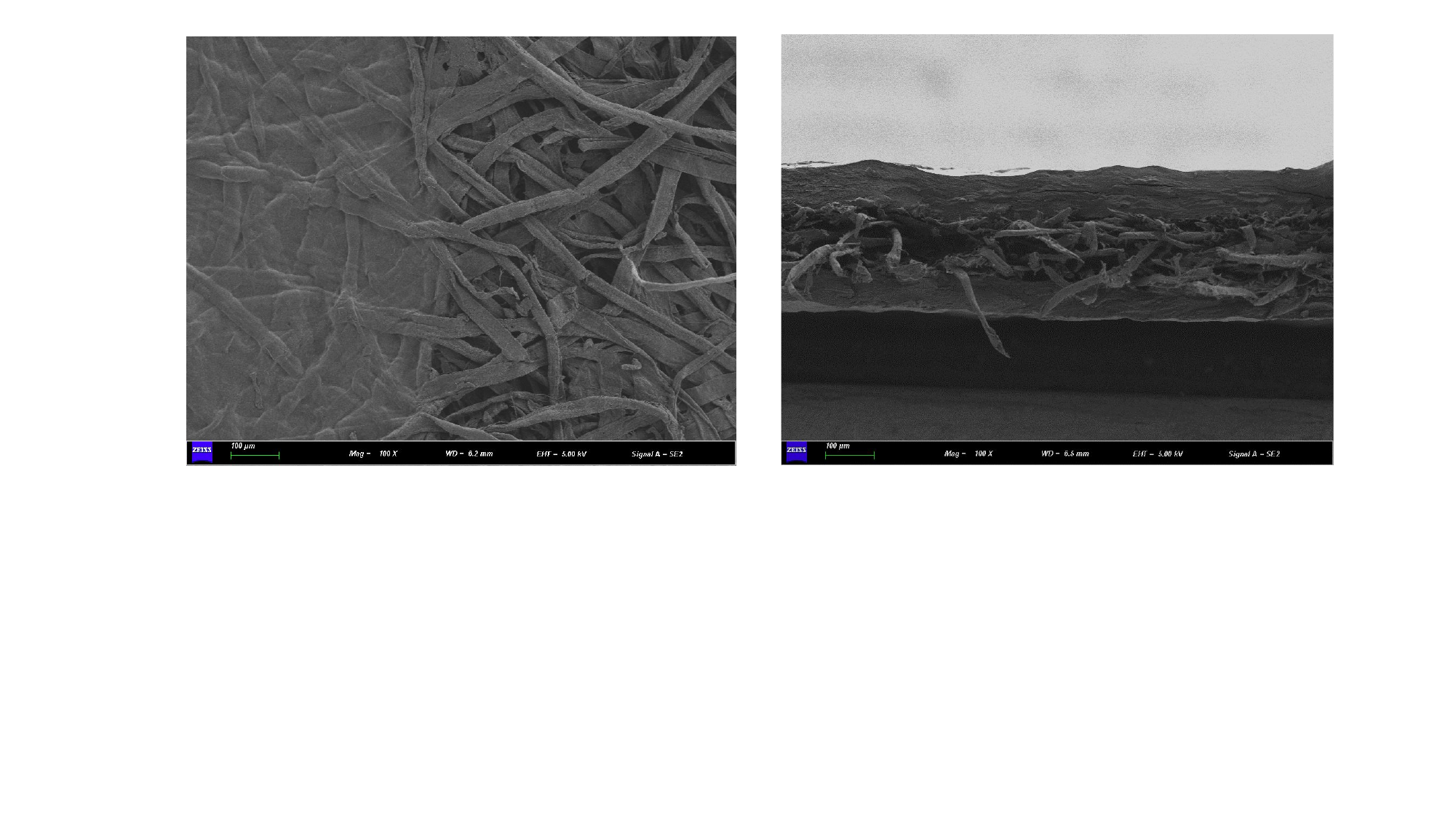

Supplement: Supplementary file 3 — Supplementary Zip [file ADVS-13-e06157-s002.zip › Supplementary Material 3/Fig2b-c.pptx]

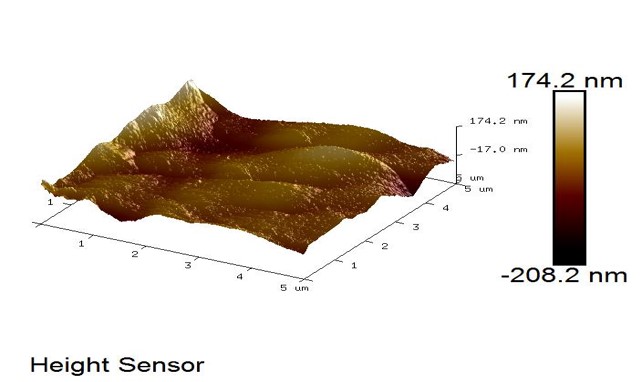

Supplement: Supplementary file 3 — Supplementary Zip [file ADVS-13-e06157-s002.zip › Supplementary Material 3/Fig3d/═╝ã1⁄44.jpg]

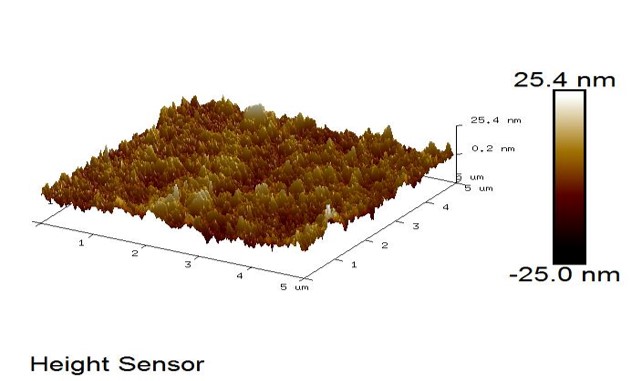

Supplement: Supplementary file 3 — Supplementary Zip [file ADVS-13-e06157-s002.zip › Supplementary Material 3/Fig3e/═╝ã1⁄45.jpg]

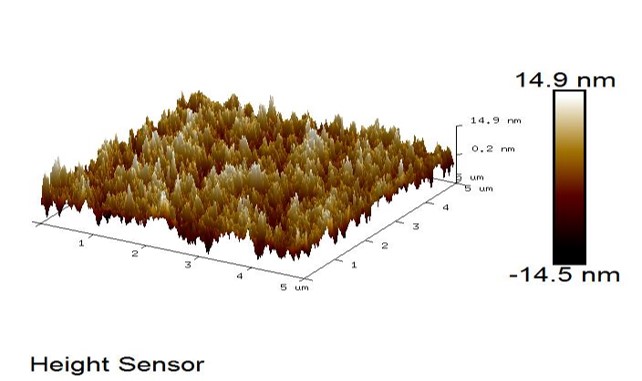

Supplement: Supplementary file 3 — Supplementary Zip [file ADVS-13-e06157-s002.zip › Supplementary Material 3/Fig3f/═╝ã1⁄46.jpg]
