# Supplementary material for: Multifunctional Thermoplastic Paper Enabled by Plant‐Cell‐Derived Additives: A Paradigm of Paper‐Based “Modern Alchemy”
Source: Adv Sci (Weinh). 2025 Nov 5;13(2):e06157. doi: 10.1002/advs.202506157 (PMC12786277; doi:10.1002/advs.202506157)
Supplement: Supplementary file 4 — Supplementary Information [file ADVS-13-e06157-s001.pdf]

Manuscript Verification Checklist (First Author – English Version)

First Author (Student) Name: Xiaoyan Yu

Manuscript Title: Multifunctional Thermoplastic Paper Enabled by Plant-Cell-Derived Additives: A Paradigm of Paper-Based "Modern Alchemy"

Journal: Advanced Science

Please review each item carefully, check the corresponding box when completed, and print and sign the document.

1. Data Authenticity

☑ The manuscript includes editable original files for all figures and tables, along with corresponding experimental data.

☑ All data presented in the manuscript are original and obtained under experimental conditions, without any modification.

2. Manuscript Integrity

☑ All images in the manuscript and its supplementary materials have been verified as correct and original, with no misuse.

☑ All elements of figures and tables are original, independently designed, and do not contain copied elements.

☑ The experimental process is comprehensively and accurately described, with major experimental methods and key phenomena documented via video.

3. Academic Integrity Statement

I solemnly affirm that all experimental data presented in this manuscript are authentic and reliable, and that all figures and tables have not been misleadingly altered.

First Author's Signature: Xiaoyan Yu

Date: 2025.07.12

Manuscript Verification Checklist (First Author – Chinese Version)

第一作者稿件（研究论文）检查核对清单

第一作者（学生）姓名：于笑颜

稿件标题：Multifunctional Thermoplastic Paper Enabled by Plant-Cell-Derived Additives: A Paradigm of Paper-Based “Modern Alchemy”

期刊：Advanced Science

请逐项确认，并在完成后勾选相应方框，并打印后签名确认。

1. 数据真实性

☑ 发表的论文中已提供论文图、表的可编辑原始文件及其对应的实验数据。

☑ 论文中的所有数据均为在实验条件下的原创数据，未作任何改动。

2. 论文完整性

☑ 论文正文及附件中的所有图片已核对无误，均为原创，不存在误用。

☑ 论文图、表的各要素均为原创，未复制任何要素，均为自行构思后独立设计后的产物。

☑ 实验过程已全面准确描述，主要实验方法及主要现象已提供视频。

3. 学术诚信声明

本人郑重承诺，该论文所有实验数据均真实可靠，所有图表均未作任何误导性修改。

第一作者签名：于笑颜

日期：2025.07.12
